# Altered expression of proteins involved in metabolism in LGMDR1 muscle is lost in cell culture conditions

**DOI:** 10.1186/s13023-023-02873-5

**Published:** 2023-10-10

**Authors:** Anabel Rico, Andrea Valls, Garazi Guembelzu, Margarita Azpitarte, Ana Aiastui, Mónica Zufiria, Oihane Jaka, Adolfo López de Munain, Amets Sáenz

**Affiliations:** 1 Neurosciences Area, Biodonostia Health Research Institute, San Sebastián, Spain; 2 CIBERNED, CIBER, Spanish Ministry of Science and Innovation, Carlos III Health Institute, Madrid, Spain; 3 Cell Culture, Histology and Multidisciplinary 3D Printing Platform, Biodonostia Health Research Institute, San Sebastián, Spain; 4 Department of Neurology, Donostialdea Integrated Health Organization, San Sebastián, Spain; 5 Department of Neurosciences, University of the Basque Country UPV-EHU, San Sebastián, Spain; 6 Faculty of Medicine, University of Deusto, Bilbao, Spain

**Keywords:** LGMDR1, Calpain 3, Cell culture, Metabolism, Myoblast, Myotubes, CD56−, FRZB, GLUT5, HKII, MCT1

## Abstract

**Background:**

Limb-girdle muscular dystrophy R1 calpain 3-related (LGMDR1) is an autosomal recessive muscular dystrophy due to mutations in the *CAPN3* gene. While the pathophysiology of this disease has not been clearly established yet, Wnt and mTOR signaling pathways impairment in LGMDR1 muscles has been reported.

**Results:**

A reduction in Akt phosphorylation ratio and upregulated expression of proteins implicated in glycolysis (HK-II) and in fructose and lactate transport (GLUT5 and MCT1) in LGMDR1 muscle was observed. In vitro analysis to establish mitochondrial and glycolytic functions of primary cultures were performed, however, no differences between control and patients were observed. Additionally, gene expression analysis showed a lack of correlation between primary myoblasts/myotubes and LGMDR1 muscle while skin fibroblasts and CD56− cells showed a slightly better correlation with muscle. *FRZB* gene was upregulated in all the analyzed cell types (except in myoblasts).

**Conclusions:**

Proteins implicated in metabolism are deregulated in LGMDR1 patients’ muscle. Obtained results evidence the limited usefulness of primary myoblasts/myotubes for LGMDR1 gene expression and metabolic studies. However, since *FRZB* is the only gene that showed upregulation in all the analyzed cell types it is suggested its role as a key regulator of the pathophysiology of the LGMDR1 muscle fiber. The Wnt signaling pathway inactivation, secondary to FRZB upregulation, and GLUT5 overexpression may participate in the impaired adipogenesis in LGMD1R patients.

**Supplementary Information:**

The online version contains supplementary material available at 10.1186/s13023-023-02873-5.

## Background

Limb girdle muscular dystrophy R1 calpain 3-related (LGMDR1), due to mutations in the CAPN3 gene, is one of the most common autosomal recessive limb-girdle muscular dystrophies. It is characterized by progressive proximal muscle weakness. The age at onset in most patients is adolescence. Loss of independent ambulation usually occurs after around 25 years of progression [1, 2]. A specific pattern of fatty substitution involving predominantly the hip adductors and hamstrings in lower limbs has been described [3].

Calpain 3, a muscle-specific protease, was first described in 1989 [4], but its role in muscle is not completely clear. In addition to its proteolytic function, calpain 3 has other functions such as being part of the sarcomere through its binding to titin [5, 6], participating in membrane homeostasis [7, 8] and in the regulation of Ca^2+^ [9].

The Wnt and mTOR signaling pathways are required for correct cellular homeostasis. In the Wnt/β-catenin signaling pathway [10], binding of Wnt ligands to Frizzled receptor inhibits the axin/GSK-3β/APC complex, leading to β-catenin accumulation [11, 12]. Stabilised β-catenin translocates to the nucleus and interacts with members of the T cell factor/Lymphoid enhancer factor (TCF/LEF) family of transcription factors to activate specific target genes [13, 14]. Without Wnt stimulation, the axin/GSK-3β/APC complex promotes the degradation of β-catenin through its phosphorylation [15]. Muscle is an exceptionally dynamic system and, therefore protein synthesis and degradation must be tightly controlled. For that purpose, negative regulators of the Wnt signaling pathway are also required, namely the Dickkopf (DKK) family and secreted frizzled-related proteins (sFRPs) [16]. Activation of the Wnt signaling pathway enhances myogenesis and inhibits adipogenesis in cultured mesenchymal stem cells [17]. Blocking the β-catenin pathway reduces myogenesis [18–20]. The Akt/mTOR signaling pathway is also considered one of the main mediators for protein biosynthesis [21]. Various molecules, including insulin, glucose, and many growth factors and cytokines can initiate Akt/mTOR signaling [22]. Akt is a vital messenger in this pathway. Activated Akt activates various downstream substrates such as protein kinases, E3 ubiquitin ligases, metabolic enzymes and transcription factors [23]. Phosphorylated Akt can phosphorylate mTOR to finally downstream activate S6K-1 and 4EBP-1, which serve as regulators of cell cycle progression or angiogenesis by enhancing translation of mRNAs [24, 25].

Furthermore, it is known that Wnt/β-catenin and Akt/mTOR signaling pathways are implicated in metabolic functions. In the aerobic glycolysis, Wnt/β-catenin pathway stimulates PI3K/Akt pathway and then HIF-1α which activates glycolytic enzymes such as glucose transporter (Glut), and monocarboxylate lactate transporter (MCT-1), among others [26–31]. Akt is essential for glucose metabolism in muscle [32], it plays a role in directing glucose transporter GLUT4 to the plasma membrane and participates in glycogen and lipids synthesis [33, 34]. Moreover, Akt regulates the mTOR signaling pathway [32] and this regulation affects the expression of many genes involved in metabolism [35, 36].

It has been established that in LGMDR1 patients, these signaling pathways are altered: (a) sFRP3 (FRZB), a specific antagonist of Wnt1, Wnt5, Wnt8 and Wnt9 [37–40], is overexpressed in LGMDR1 patients [41, 42]; and (b) the expression of mTOR, as well as its phosphorylated form and the phosphorylation of proteins downstream of this pathway is severely reduced in the muscles of LGMDR1 patients [43].

Based on our previous findings, metabolic function could also be altered in LGMDR1 patients, thus we proceeded to study the proteins involved in mitochondrial function and in glycolysis. The results obtained in muscle showed alterations in metabolism regulating key proteins. On the contrary, no differences in metabolic functions and in gene expression analysis in primary cultures between LGMDR1 patients and controls has been observed.

## Results

### The expression of proteins involved in metabolism is altered in the muscle of LGMDR1 patients

Proteins related to glucose metabolism and mitochondrial function were studied in muscle from controls and LGMDR1 patients. GLUT5 (a fructose transporter) [44, 45] and MCT1 (a lactate/pyruvate transporter) [46] were analyzed because their genes (*SLC2A5* and *SLC16A1* respectively) showed expression upregulation in the muscle of the patients in a previous study [41]. In addition, other proteins that participate in cell metabolism were analyzed: hexokinase II (phosphorylates glucose to produce glucose-6-phosphate) [47–51] and PGC1α (regulator of mitochondrial biogenesis, mtDNA copy number, mitochondrial fission, mitophagy, fat and carbohydrate metabolism and angiogenesis in skeletal muscle [52–55]. Moreover, due to their regulatory role in the mTOR pathway, Akt and P-Akt (S473) were analyzed.

**Fig. 1:**
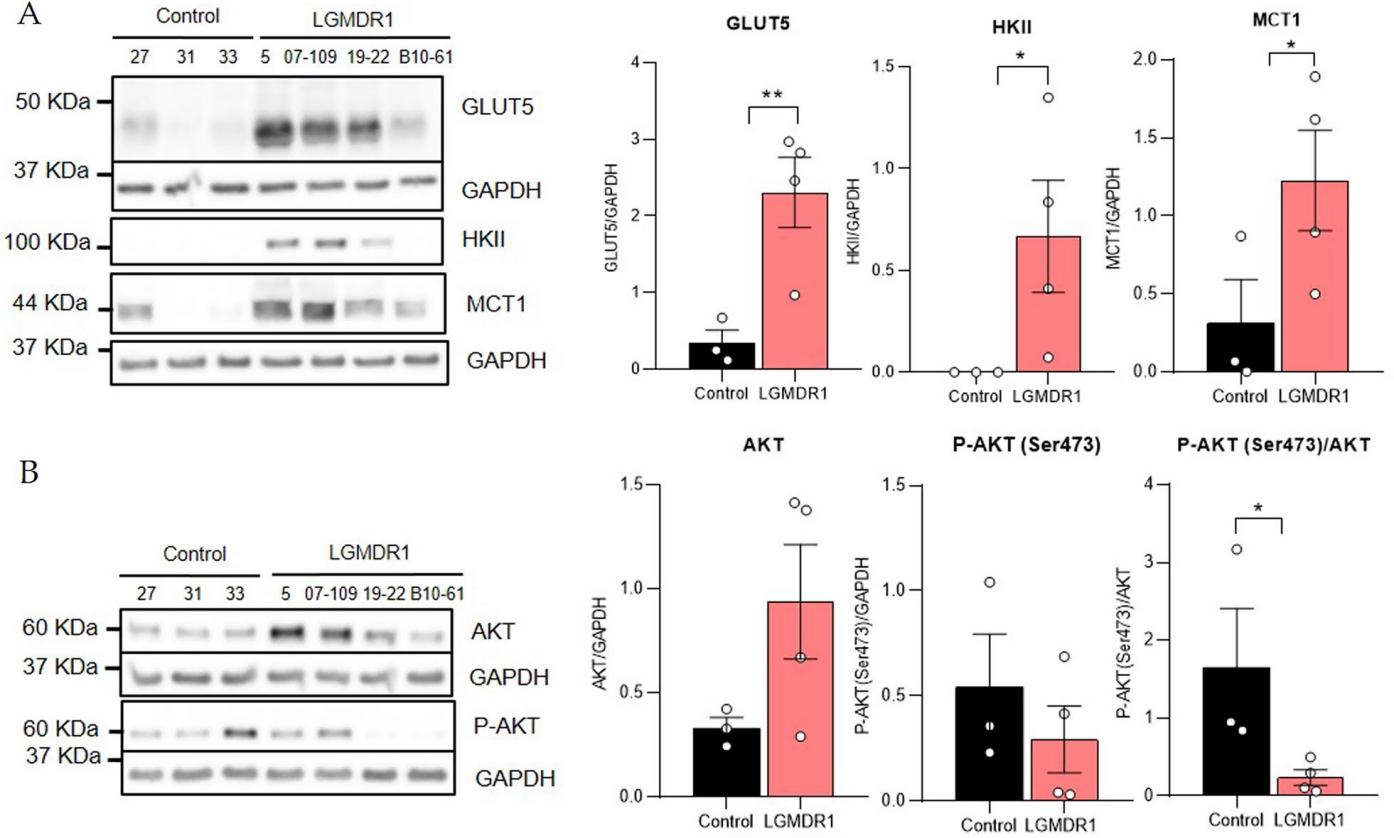
Protein expression analysis in muscle samples. Western blot and densitometry analysis of **A** GLUT5 (*p* = 0.0086), HKII (*p* = 0.0481) and MCT1 (*p* = 0.0479) and **B** AKT and P-AKT (S473) (*p* = 0.0400 for P-AKT (S473)/AKT ratio) in samples from muscles of controls and LGMDR1 patients. Error bars represent standard error of the mean (SEM). The statistical significance was assessed by an unpaired T-test

Increased expression of GLUT5, HKII and MCT1 proteins in the muscles of LGMDR1 patients was found (Fig. 1A). On the other hand, a slight increasing trend in PGC1α expression was found (Additional file 1: Fig. S1). Finally, Akt phosphorylation rates showed a significant decrease in LGMDR1 patients’ muscles.

### Myoblasts/myotubes of LGMDR1 patients compared to controls do not show differences in mitochondrial and glycolytic functions

Since alteration in the expression of proteins involved in metabolism had been observed in the muscle of LGMDR1 patients, we proceeded to study the metabolic processes, mitochondrial and glycolytic function, in primary cultures.

In the mitochondrial function study, both, in myoblasts and in myotubes at day 10 of differentiation, no differences in oxygen consumption were observed between patients and controls (Additional file 2: Fig. S2).

In the glycolytic function, in patients’ myoblasts, a slight upward trend was observed compared to controls that did not reach statistical significance. In the myotubes, no differences were observed between patients and controls (Additional file 3: Fig. S3).

Finally, different biochemical parameters related to mitochondrial or glucose metabolism in the culture media of myoblasts and myotubes at day 11 of differentiation of two healthy controls and two LGMDR1 patients were studied. No differences were detected between controls and patients in any of the studied parameters: lactate (mmol/l), ammonium (µmol/l), lactate dehydrogenase, LDH (U/l); pH, calcium, Ca^+2^ (mg/dl) and glucose (mg/dl) (data not shown).

### Primary cultures of controls and LGMDR1 patients tend to lose the observed differences in the muscles

The expression of PGC1α, MCT1 and hexokinase II proteins was also analyzed in myotubes at day 10 of differentiation and no expression differences between patients and controls were observed (data not shown).

Moreover, since myoblasts/myotubes cell cultures are a dynamic system, expression profiling at different days (day 8, 10 and 20 of differentiation) was added to the previously performed analysis of the myoblasts and myotubes at day 16 of differentiation [42] (the analyzed 63 genes are shown in Additional file 4: Table S1). In all the analyzed points (at 8, 10 and 20 days of differentiation) the genes that presented differential expression were mostly downregulated.

The myotubes at day 8 of differentiation showed the highest number of genes whose expression correlated with muscle. The genes that showed downregulation consistent with that observed in muscle (fold change value lower than 0.5) were: *CA2*, *EGR1*, *FOS*, and *SMG1*.

At day 10 of differentiation, few differences were detected between controls and patients. The *ALDH2* gene was the only gene that showed the same tendency as in muscle. Finally, at day 20 of differentiation *EGR1* showed correlation with the expression in muscle, although *FRZB* almost reached a fold change value of 2 (Additional file 4: Table S1). The expression of the *FRZB* gene in particular, at day 10 of differentiation showed an increasing trend (1.52) that later, at day 20 of differentiation (1.98) was maintained (Additional file 4: Table S1). In the myotubes at day 16 of differentiation the expression of FRZB protein was increased [42]. However, none of the mentioned expression differences, except for EGF1 (MT8), were statistically significant.

**Fig. 2:**
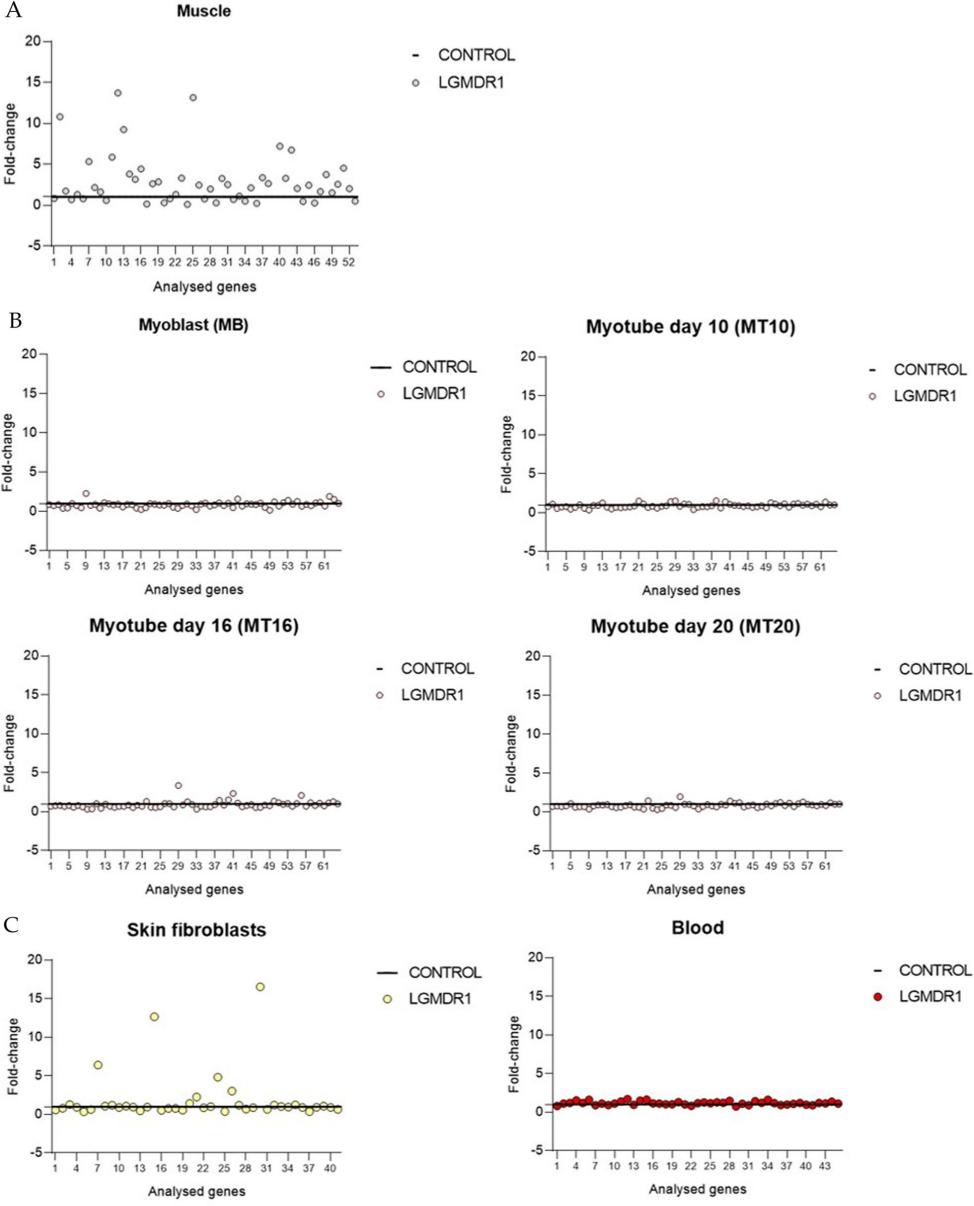
Comparison between gene expression analysis in LGMDR1 patients and controls. **A** In muscle (patients n = 3 and controls n = 3) **B** in myoblasts (MB)/myotubes (MT) at day 10, 16 and 20 of differentiation (patients n = 4 and controls n = 3) and **C** in skin fibroblasts (patients n = 3 and controls n = 2) and in blood (patients n = 5 and controls n = 5). Each dot represents the analysed 63 genes

qRT-PCR results were also analysed to better define the correlation of the expression between patients and controls in muscle and in vitro analyses. The expression of the analysed genes was similar between controls and patients in myoblasts and myotubes (Fig. 2A).

### FRZB is overexpressed in more advanced stages of differentiation in myotubes from LGMDR1 patients

**Fig. 3:**
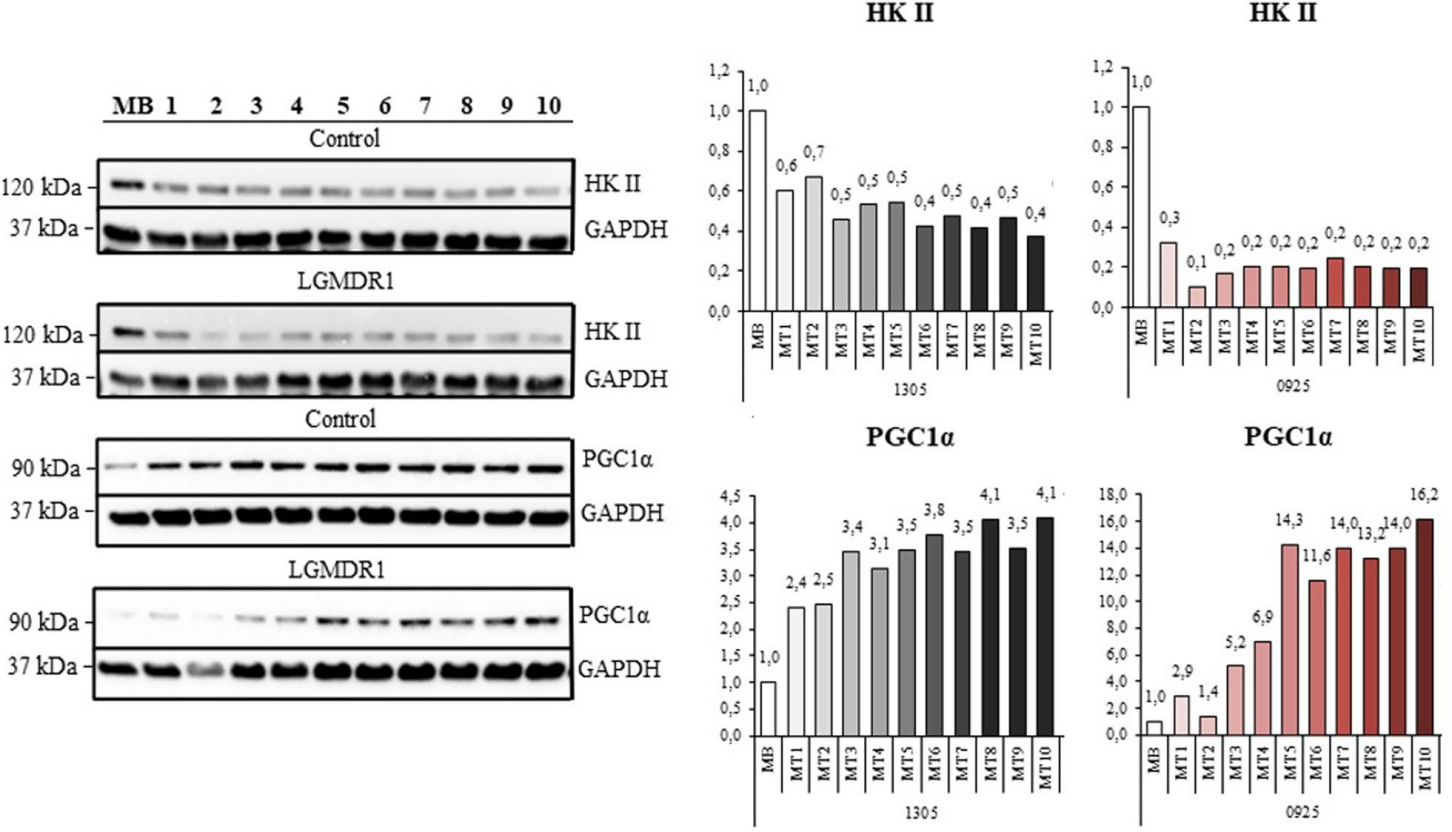
Western blot analysis of hexokinase II and PGC1α expression. One control (black, 13–05) and one LGMDR1 patient (red, 09–25) during myogenesis (from myoblasts to myotubes at 10 days of differentiation). The gradient of colour in both groups represents the increasing differentiation status

On the other hand, it is known that the predominant metabolism depends on the cell type, and myoblasts show a more glycolytic metabolic profile than myotubes [56]. Thus, to establish the predominant metabolism of myoblasts and myotubes in different stages of differentiation, the levels of hexokinase II (HKII) and PGC1α in the myogenesis of a control and a patient were evaluated. The results show that hexokinase II levels decrease as differentiation progresses (Fig. 3). Contrary to what occurs with hexokinase II, PGC1α levels increase as differentiation progresses. Similar tendencies have been observed between patients and controls in the expression of these proteins.

**Fig. 4:**
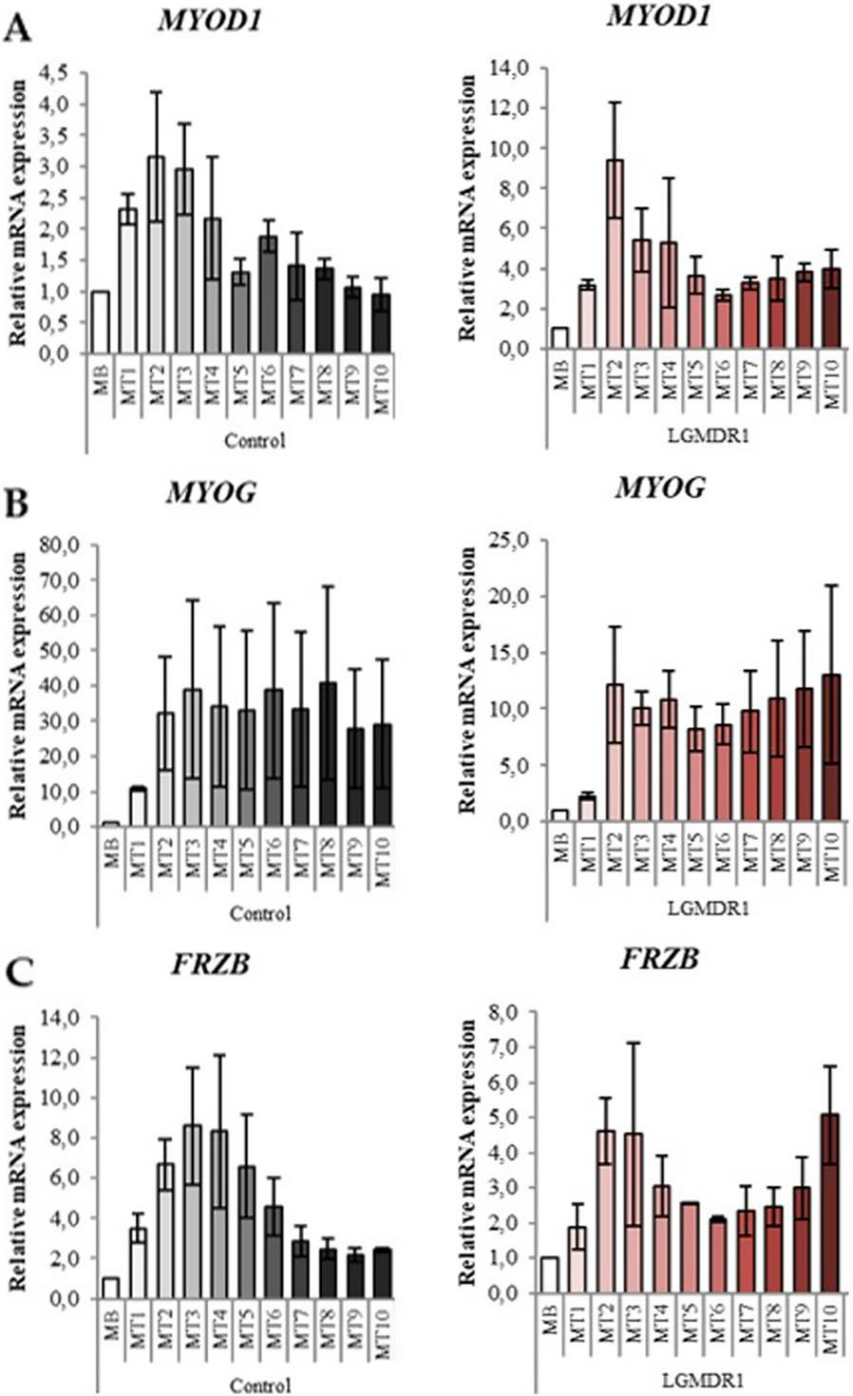
Gene expression during myogenesis, from myoblasts (MB) to myotubes (MT) at day 11 of differentiation. **A** *MYOD1*, **B** *MYOG* and **C** *FRZB*. Means of two controls (black) and two patients (red) for each gene have been analyzed. The gradient of color in both groups represents the increasing differentiation status. *GAPDH* used as normalization gene. For each control and patient, myoblasts value was normalized to 1. Bars represent standard error of the mean (SEM)

As mentioned, given the absence of correlation of the differentiated myotubes with the muscle of LGMDR1 patients, the expression in the differentiation from the myoblast stage to myotube at day 10 was analyzed uninterruptedly in two controls and two patients (Fig. 4). First, genes *MYOD1* and *MYOG*, whose participation in myogenesis has been widely established [57], were analysed. It is observed that *MYOD1* presents a significant increase from the beginning of differentiation, reaching maximum expression on day 2 of differentiation in both patients and controls (Fig. 4A).

For *MYOG* expression, a maximum is observed between days 2–3 of differentiation, it is maintained and it is not reduced to the initial levels detected in myoblasts. A similar pattern is observed in patients and controls (Fig. 4B).

Since FRZB is the gene that has shown the best correlation in muscle and myotubes at different days of differentiation, it was also analyzed in the differentiation process. In all cases, a maximum expression of FRZB is observed between days 2 and 3 of differentiation; however, in patients, an additional increase in the expression from days 9–10 of differentiation was detected, reaching again a maximum expression at day 10 (Fig. 4C).

### CD56− cells and skin fibroblasts present better correlation with muscle

**Fig. 5:**
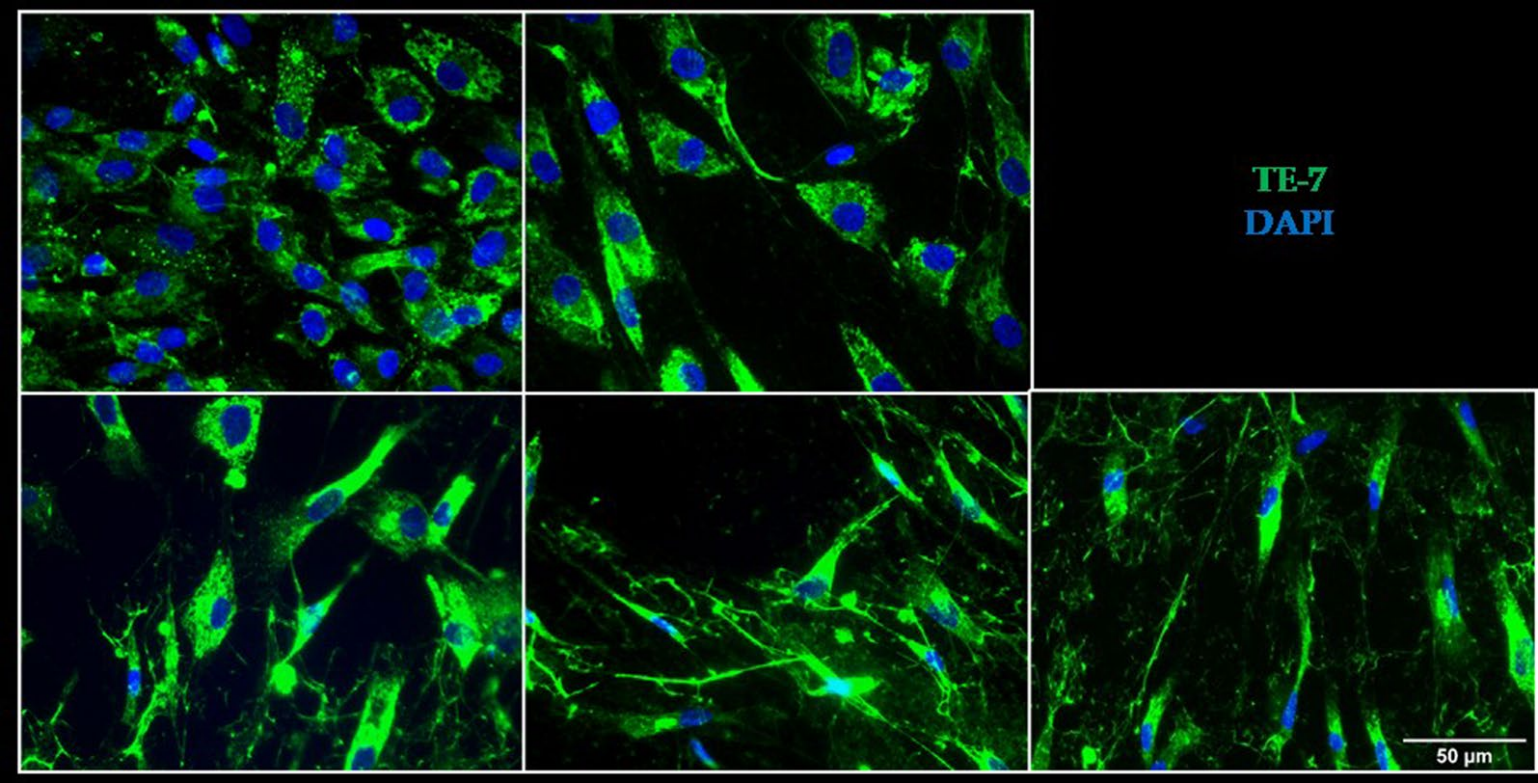
Immunoflorescence of cultured CD56− cells. Two controls (13–09 and 15–12) and three patients (10–39, 09–25 and 09–21). Green: TE-7 antibody. Blue: DAPI

CD56− cells were used in order to investigate the origin of the fibrosis or the fat tissue substitution observed in LGMDR1 patients. Human skeletal muscle fibroblasts are progenitors that can remain as extracellular-matrix-producing cells or differentiate into adipocytes [58]. In the immunofluorescence analysis, altered morphology of CD56− cells was observed in cells obtained from patients’ muscles (Fig. 5).

Thus, some of the deregulated genes implicated in fibrosis in muscle were analysed in these cells. *FRZB*, *ITGB1BP2*, *MHY3*, *MYOM3* and *TFRC* genes showed increased expression tendency. *RORA* and *SLC16A1* genes were also analyzed and showed a tendency to increase expression in CD56− cells of patients, but only in *ITGB1BP2* and *MYOM3* the increase was statistically significant (*p* < 0.5) (Additional file 4: Table S1).

On the other hand, it is becoming difficult to collect muscle biopsies in patients (as diagnoses are increasingly being made without the need for a biopsy) and it is difficult to make comparisons between samples that are heterogeneous in terms of muscle type, patient’s age and sex. These factors make it important to attempt to standardize research in this field using alternative cell models and a clinical setting. In peripheral blood there are incomplete isoforms of calpain3 expressed at the RNA level [59, 60] and skin biopsies are less invasive approaches than muscle biopsies. For these reasons, we selected these tissue and cells to assess whether the results previously found in muscle from patients with calpain 3 deficiency can be replicated and thereby enable these cultures to be considered as alternative cell models for the physiological analysis of LGMDR1.

We also analyzed 63 genes that had previously showed altered expression in muscle of LGMDR1 patients (Additional file 4: Table S1) [41, 61]. Skin fibroblasts showed deregulation of some gene in LGMDR1 patients. The pattern of deregulation of gene expression was not always the same; that is, in some cases, the expression was upregulated in muscle and downregulated in skin fibroblasts and, in other cases, vice versa (Additional file 4: Table S1). Nevertheless, there were several genes that showed concordant overexpression in patients’ skin fibroblasts and muscle, namely *CD9, ITGB1BP2*, *FRZB* and *IGF1* (Additional file 4: Table S1). Only *FRZB* gene expression showed statistical significance. In peripheral blood, no expression was detected for 18 out of the 63 genes studied either in controls or in LGMDR1 patients. Amongst the genes that were expressed in blood, no differences were detected between the samples from LGMDR1 patients and controls (Additional file 4: Table S1).

When analyzing the correlation between the expression of the genes of the controls and the patients, the lowest correlation was shown by skin fibroblasts with the exception of muscle (Fig. 2).

Related to the *CAPN3* transcript variants expressed in these cell types, adult skeletal muscle only express the full-length variant. In a previous study of our group, it was described that white blood cells (WBC) expressed different transcript variants with three in frame deletions. Exon 15 is systematically spliced out and exon 6 and 16 could be present or absent giving rise to four different variants in WBC [60]. However, expressed *CAPN3* transcript variants in CD56− and fibroblasts could not been determined.

### Activation of the Wnt signaling pathway increases glycolysis in myoblasts

**Fig. 6:**
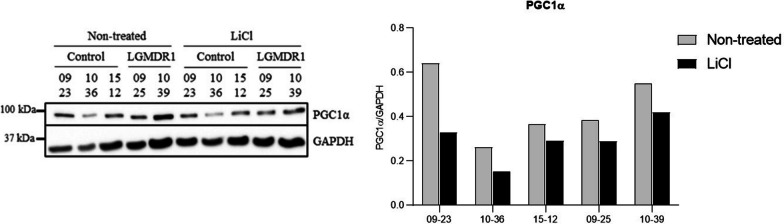
PGC1α quantification in treated and not treated myoblasts. **A** Western Blot analysis **B** Densitometry. Controls n = 3 (09–23, 10–36 and 15–12) and patients n = 2 (09–25 and 10–39). The statistical significance was assessed by a paired T-test (*p* = 0.0287)

Although no differences between patients and controls at the cellular level in glycolysis or mitochondrial function were observed, we wanted to analyze the effect of the activation of the Wnt pathway by means of lithium. In myoblasts, the expression of PGC1α was significantly reduced after treatment with lithium (Fig. 6). In myotubes treated with lithium, no changes were observed in the expression of PGC1α (data not shown).

**Fig. 7:**
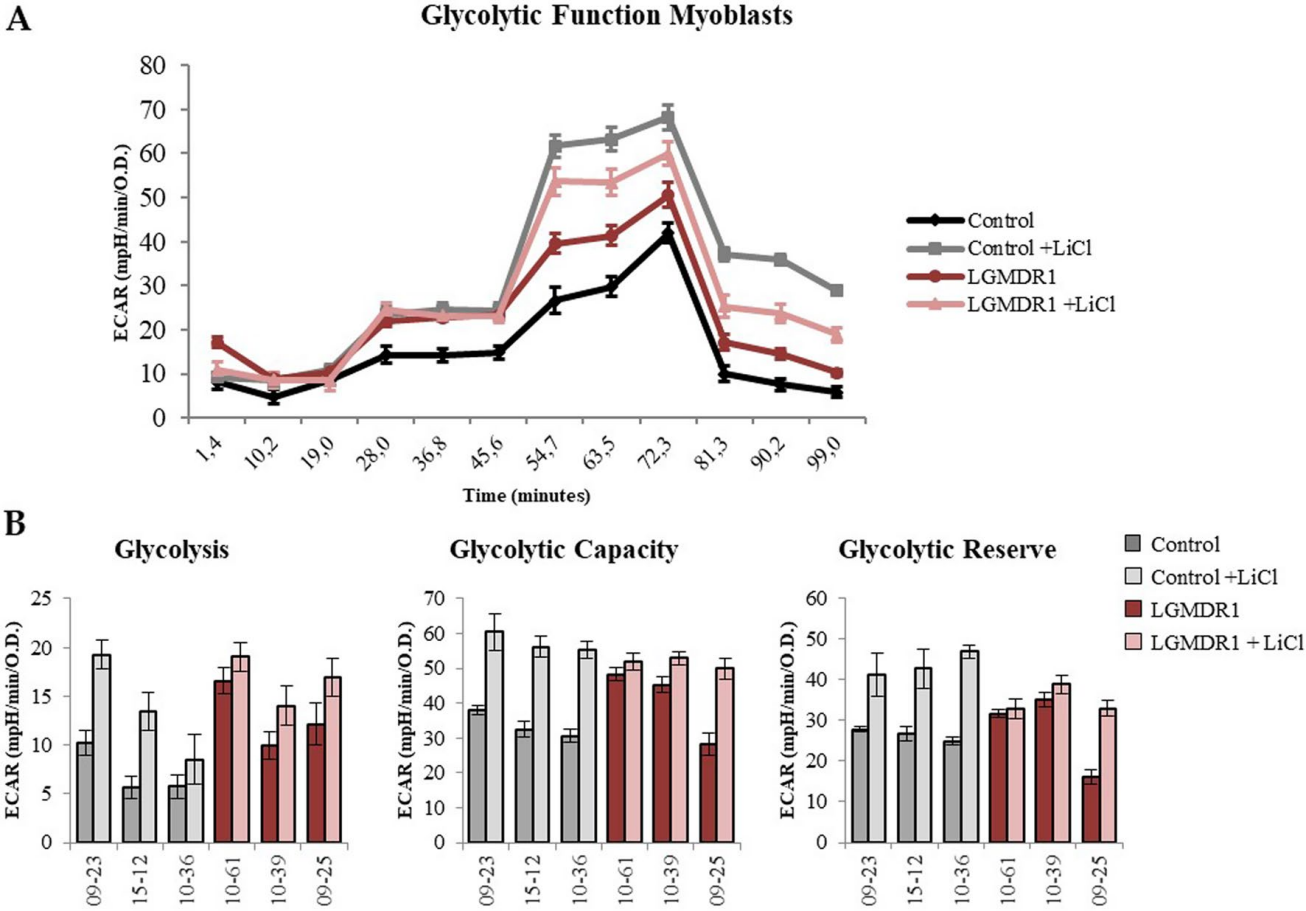
Glycolytic function assay in treated and not treated myoblasts. Controls n = 3 (09–23, 15–12 and 10–36) and patients n = 3 (10–61, 10–39 and 09–25). **A** Glycolytic function assay representation. **B** Analyzed parameters: Glycolysis, Glycolytic Capacity and Glycolytic Reserve (Error bars represent standard error of the mean (SEM) of technical replicates, n = 6)

When analyzing the glycolytic function, it was observed that lithium caused an increase in Glycolysis, Glycolytic Capacity and consequently in the Glycolytic Reserve in myoblasts, this being more pronounced in controls than in LGMDR1 patients (Fig. 7). In contrast, in myotubes, lithium administration did not show any change (data not shown).

## Discussion

### Lack of correlation between muscle and cells in culture

Since the data shown here, due to sample availability, were not obtained from the same patient and control in muscle and cell cultures, these results should be treated with caution. Nevertheless, the lack of correlation between muscle and cells in differentiation, as well as the absence of significant differences found in the analysis of metabolic functions (mitochondrial and glycolytic) and the results of the analysis of biochemical parameters, underlines that, there are physiological differences between culture and in vivo in muscle.

Cultured myotubes lack the in vivo microenvironment and the communication that exist with other cells through direct contact and via bioactive substances [62]. Thus, the observed pattern could be impaired among other factors by the lack of the extracellular matrix (ECM), as cell adhesion to the ECM generates various signals that regulate important physiological events, including cell spreading, migration and growth [63, 64].

Differences found between controls and patients in muscle, were lost between myoblasts/myotubes of controls and LGMDR1 patients. However, contrary to our finding, correlation of expression patterns between muscle and primary cell cultures (myoblasts and skin fibroblast) in other muscular dystrophies, such as Myotonic Dystrophy type 1 has been reported [65].

Nevertheless, loss of differences in vitro has also been described previously in other works [42, 66–68]. Specifically, LaFramboise and colleagues [69] compared the expression of MyHC isoforms between primary cultures obtained from different muscles, observing that all cells form similar uniform differentiated cells.

Additionally, in 2010 Raymond and colleagues compared expression profiling between human cultured myotubes and skeletal muscle tissue. Downregulation in the expression profile of genes associated to mitochondria, involved in metabolism and muscle-system/contraction process was detected in cell cultures. Specifically, among the most downregulated gene expression, that of the *CAPN3* gene was found [70]. This relevant finding might be one of the main factors that impacts in the cellular models, making the control profile more similar to the LGMDR1 patient’s profile.

It is known that the current best in vitro model of myoblast maturation is the 3D culture, although this approach requires special laboratory equipment [36]. However, Brunetti and colleagues [71] have proposed an affordable 2D myoblast maturation culture. They achieved myotubes with acetylcholine receptor clusters, striation and adult isoforms expression of skeletal muscle proteins.

Despite the limitations of primary myotubes, these are and have been very useful models for LGMDR1 since they allowed the identification of relevant differences in the patients’ myotubes, such as abnormal fusion of myoblasts, absence of the integrin β1D isoform substitution and the increase in the expression of *FRZB* gene among others [42]. In addition, their use for the physiology studies after drug administration are valuable, as recently demonstrated in a study carried out by our group [43].

### Wnt signaling inactivation possible cause of increased adipogenesis in LGMDR1

Myoblasts have a more glycolytic profile [56] and the activation of the Wnt pathway, by means of Li administration, leads to a reduction in the expression of PGC1α and an increase in glycolysis (Figs. 6 and 7). This has also been observed in adipocytes, where activating the Wnt signaling pathway inhibits the expression of the PPARγ coactivator-1α gene, encoding PGC1α protein [72]. Activation of the Wnt signaling pathway enhances myogenesis and inhibits adipogenesis in cultured mesenchymal stem cells [17]. Both, muscle cells and adipocytes, are derived from mesenchymal stem cells. Most of these cells develop into myogenic cells, but a small portion of them differentiate into adipocytes which are the basis for intramuscular fat [20]. Moreover, human skeletal muscle fibroblasts, CD56−, are progenitors that can remain as extracellular-matrix-producing cells or differentiate into adipocytes [58]. It has been stablished that expression of inhibitors of the Wnt signaling pathway such as secreted frizzled related proteins (sFRP1, sFRP2 and sFRP3 = FRZB) causes spontaneous adipocyte conversion in mouse preadypocytes [73]. Thus, the upregulation of *FRZB* gene expression in LGMDR1 CD56− cells, inactivating Wnt signaling pathway, could be one of the possible causes of the fatty tissue substitution in the patients’ muscle.

### Wnt, mTOR signaling pathways and metabolism in LGMDR1 patients’ muscle

**Fig. 8:**
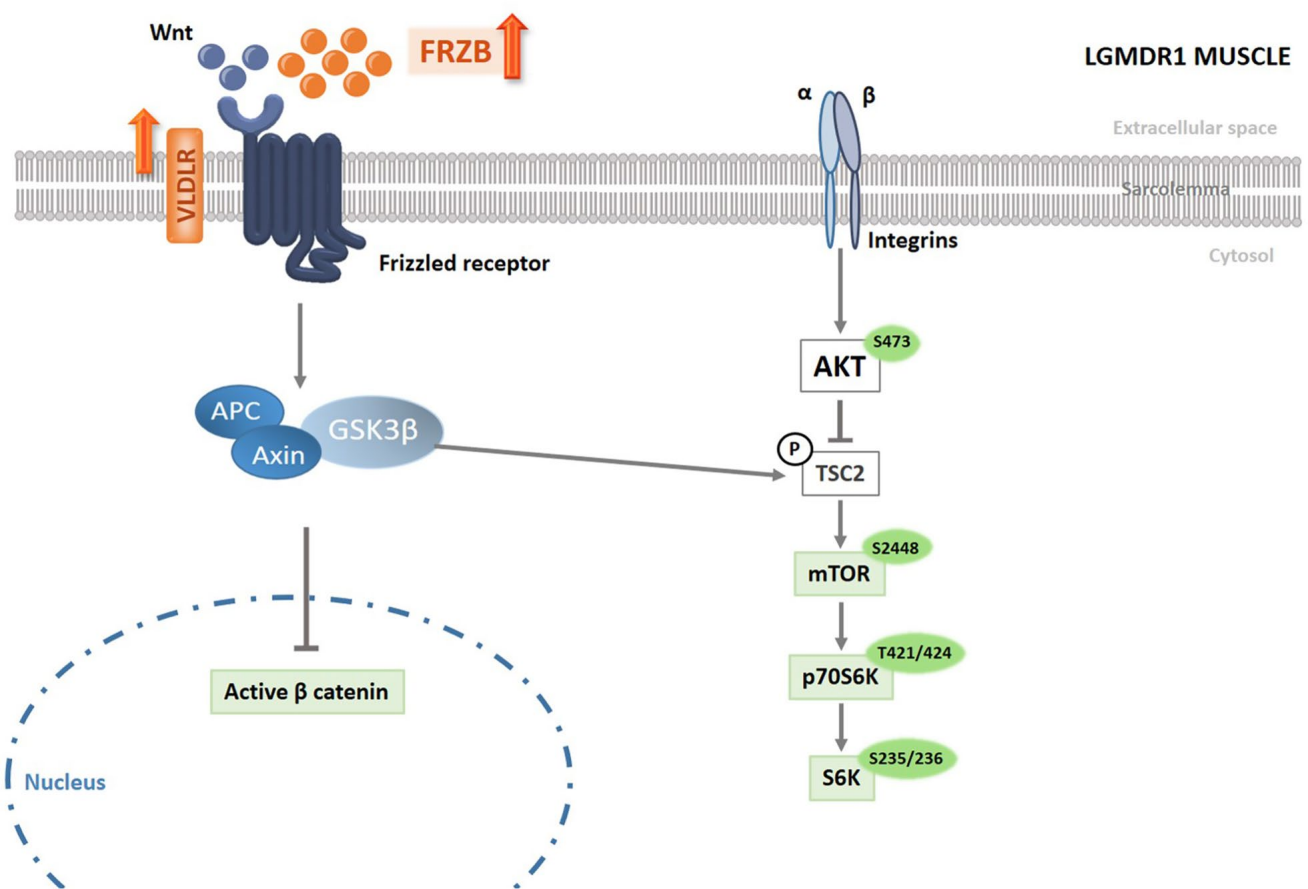
Wnt and mTOR signaling pathways. Schematic representation of the altered protein expression or phosphorylations in Wnt and mTOR signaling pathways in LGMDR1 muscle. Orange: Upregulated expression in LGMDR1. Green: Downregulated expression or phosphorylation in LGMDR1

Wnt pathway activity is reduced in LGMDR1 patients muscle due to the increased expression of its inhibitors, FRZB (sFRP3) and VLDLR [41, 42] and the reduction in active β-catenin [43] (Fig. 8). Relationship between Wnt signaling pathway regulation and metabolism has already been described and FRZB could be an important player in this interaction. In our work, even the observed lack of correlation between the muscle and the cells, *FRZB* shows overexpressing trend in patients’ myotubes (Fig. 4C and Additional file 4: Table S1). It is known that FRZB participates in metabolism regulation by means, at least, of these two genes: *MYOD*, that regulates the oxidative metabolic capacity of adult skeletal muscle [74], and *SLC16A1*, that encodes MCT-1, a pyruvate transporter that can modulate the relative levels of glycolysis and oxidative phosphorylation [33, 75]. FRZB has been reported to inhibit myogenesis by reducing MyoD expression [37, 76]. This is also likely occurring in LGMDR1 patients’ muscle, where *FRZB* expression is increased and *MYOD1* expression is reduced [41]. Furthermore, in human myotubes when *FRBZ* is silenced, the expression of *MYOD1* increases [77]. *SLC16A1,* a direct Wnt target gene, is coordinately regulated with other genes that promote glycolysis in colon cancer cells [33] and it is downregulated in Frzb^−/−^ mouse model [77]. All these findings supports that FRZB plays an important role in the metabolism and in the pathophysiology of LGMDR1.

Related to the mTOR signaling pathway, in LGMDR1patients’ muscles there is a reduction in mTOR protein and in the phosphorylation of proteins downstream of the pathway [43] (Fig. 8). The inactivation of this pathway may cause important consequences since mTOR regulation affects the expression of many genes involved in nutrient and protein metabolism [78]. A series of studies proposed the role of the mTOR-PGC-1α-mitochondria axis in the regulation of myogenesis [79]. The mTOR pathway is also regulated, among other factors, by Akt phosphorylation. The full activation of Akt requires the phosphorylation of Ser473 and Thr308 [80]. Although the regulatory mechanisms for which Ser473 phosphorylation is mediated are not well known yet, an mTOR-complex is suggested to be involved (mTORC2) [81]. A diminished Akt activity is associated with dysregulation of cellular metabolism and cell death [82]. The decrease in the Akt activity in C2C12 cells reduces the expression of genes coding HK-II, GLUT4 and PGC1α proteins [83] and in mouse muscle, it reduces the total mitochondrial content, mitochondrial function and *PGC1α* expression [84, 85].

Related to HK-II expression, many studies support the correlation between Akt activity and HK-II expression [86–93]. HK-II is a constitutively active kinase and the changes in its expression level directly impact cellular glucose metabolism [86, 94–98]. The previously described mTOR pathway impairment [43], together with the reduction in Akt phosphorylation in the LGMDR1 patients’ muscles, could induce a deregulation of the metabolism. However, since Akt phosphorylation is reduced and HKII expression increased in LGMDR1 patients’ muscle, contrary to what has been described in mouse, other factors should be considered as responsible of the HK-II overexpression. In cardiomyocytes and in vivo heart protective effect of overexpression of HK-II has been established [99–101]. Moreover, mitochondrially associated HKs (mitoHKs) can exert protective effects on mitochondria to prevent mitochondrial death pathways [102–107]. Therefore, the existence of a similar protective or compensatory effect for LGMDR1 patients’ muscle could be suggested, but further studies would be required.

Finally the expression of GLUT5, overexpressed in LGMDR1 patients´ muscle, is strictly confined to the plasma membrane of adipocytes and to the sarcolemma of skeletal muscle where it is responsible for facilitating fructose uptake from the blood into these tissues [44, 45, 108]. GLUT5 protein is predominantly expressed in type II (fast) fibers in human skeletal muscle [109]. Certainly, the role of GLUT5 in skeletal muscle is unclear, but it is known that GLUT5 is implicated in regulating adipose tissue differentiation [110]. Thus, its overexpression could be another factor involved in the fat substitution process observed in patients’ muscles.

## Conclusions

Deregulated expression of proteins implicated in metabolism was observed in LGMDR1 patients. However, metabolic functional analysis and gene expression analysis in primary LGMDR1 myoblasts/myotubes did not show correlation with muscle. Thus, these results evidence the limited usefulness of primary myoblast/myotubes for LGMDR1 studies. However, CD56—cells present a better gene expression correlation with muscle. Despite these limitations, *FRZB* is the only gene that showed upregulation in all the analyzed cell types (except in myoblasts), suggesting its role as a key regulator of the pathophysiology of the LGMDR1 muscle fiber*.*

Finally, the Wnt signaling pathway inactivation, secondary to FRZB upregulation, and GLUT5 overexpression may participate in the impaired adipogenesis in LGMD1R patients. Nevertheless further studies are required to reveal the underlying mechanisms of the disease, but the recent findings could pave the way to find possible therapeutic targets.

## Methods

### Biological samples

All participants gave informed consent before donating biological material, using forms approved by the Ethics Committee on the Use of Human Subjects in Research at Donostia University Hospital. Muscular biopsies from proximal limbs (biceps, triceps, deltoid and quadriceps), skin and blood from controls and LGMDR1 patients were obtained. It is becoming difficult to collect muscle biopsies in LGMDR1 patients since diagnoses are being made without the need for a biopsy. Moreover, as we consider it is not ethical to perform a biopsy only for research purposes in patients whose muscles have not regeneration capacity, we used the available samples of our historical patients’ series. Controls were healthy individuals that underwent surgery for bone fractures and the muscle biopsies were obtained during the surgery. All patients presented two mutations in the *CAPN3* gene. The pseudo-asymptomatic patients only showed pathological features in the biopsies. Not all the muscle samples were available for all the different later analysis; detailed availability is described in Table 1.

**Table 1:** Muscle, myoblast, CD56− cells, skin fibroblast and blood samples from healthy controls and LGMDR1 patients

Biopsy number	Status	Gender	Sample (Tissue of origin)	Age#	Functional status	*CAPN3* gene mutations	
						Mutation 1	Mutation 2
*Muscle samples*							
27	Control	Male	Quadriceps	50	–	–	–
31	Control	Male	Quadriceps	46	–	–	–
33	Control	Male	Deltoid	51	–	–	–
22–09	Control	Male	Deltoid	47	–	–	–
05	LGMDR1	Male	Deltoid	13	Pseudo-Asympt	p.(Arg788SerfsX14)	p.(Arg788SerfsX14)
09	LGMDR1	Female	Biceps	14	Pseudo-Asympt	p.(Arg490Trp)	p.(Gly691TrpfsX7)
07–109	LGMDR1	Male	*	10	Pseudo-Asympt	p.(Arg698Gly)	p.(Arg748Glu)
36**	LGMDR1	Male	Quadriceps	26	Ambulant	p.(Lys254Glu)	p.(Pro637HisfsX25)
B10-61	LGMDR1	Female	Quadriceps	23	Ambulant	p.(Pro22Glnfs*35)	p.(Lys211_Glu323del)
B09-26	LGMDR1	Female	Quadriceps	48	Non-ambulant	p.(Arg489Tyr)	p.(Arg788Ser)
19–22	LGMDR1	Female	Deltoid	25	Ambulant	p.(Arg788SerfsX14)	Complete gene deletion
*Myoblast/Myotubes and CD56− cells samples*							
09–23	Control	Male	Triceps	26	–	–	–
10–36	Control	Male	Biceps	23	–	–	–
13–05	Control	Male	Quadriceps	14	–	–	–
13–07	Control	Female	Biceps	36	–	–	–
13–09	Control	Male	Quadriceps	37	–	–	–
15–12	Control	Male	Deltoid	36	–	–	–
09–21	LGMDR1	Male	Biceps	19	Ambulant	p.(His690ArgfsX9)	p.(His690ArgfsX9)
09–24	LGMDR1	Female	Deltoid	47	Non-ambulant	p.(Arg788SerfsX14)	p.(Lys595ValfsX70)
09–25	LGMDR1	Male	Deltoid	28	Ambulant	p.(Lys254Glu)	p.(Pro637HisfsX25)
10–39	LGMDR1	Male	Deltoid	29	Non-ambulant	p.(Lys254del)	p.(X822Leuext62X)
10–61	LGMDR1	Female	Quadriceps	23	Ambulant	p.(Pro22Glnfs*35)	p.(Lys211_Glu323del)
*Skin fibroblasts*							
C19	Control	*	Skin		–	–	–
C21	Control	*	Skin		–	–	–
P1	LGMDR1	Female	Skin	47	Non-ambulant	p.(Arg788SerfsX14)	c.1782 + 1072G > C
P2	LGMDR1	Female	Skin	46	Non-ambulant	p.(Arg788SerfsX14)	p.(Arg788SerfsX14)
P23	LGMDR1	Male	Skin	15	Unknown	p.(Arg788SerfsX14)	p.(Arg788SerfsX14)
*Peripheral blood*							
AnA	Control	Female	Blood	33	–	–	–
AiA	Control	Female	Blood	34	–	–	–
AnG	Control	Female	Blood	33	–	–	–
LeC	Control	Female	Blood	26	–	–	–
PiC	Control	Female	Blood	32	–	–	–
94–121.1	LGMDR1	Male	Blood	32	Ambulant	p.(Gly222Arg)	p.(Arg788SerfsX14)
96–137	LGMDR1	Male	Blood	61	Non-ambulant	p.(Arg788SerfsX14)	p.(Arg788SerfsX14)
97–21.1	LGMDR1	Female	Blood	50	Non-ambulant	p.(Arg788SerfsX14)	p.(Arg788SerfsX14)
98–304	LGMDR1	Male	Blood	24	Ambulant	p.(Arg788SerfsX14)	p.(Arg788SerfsX14)
02–11.3	LGMDR1	Male	Blood	*	Ambulant	p.(Arg788SerfsX14)	p.(Arg788SerfsX14)

*Pseudo-Asympt.* Pseudo-Asymptomatic

*Information not available. **36, also identify as 09–25 in different blots. ^#^Age at biopsy

### Primary human skeletal muscle culture

Human proximal muscle biopsies were minced and cultured in a monolayer according to the method described by Askanas [111]. To obtain highly purified myoblasts, primary cultures were sorted by immunomagnetic selection based on the presence of the early cell surface marker CD56 (separator and reagents from Milteny Biotec). CD56+ cells and CD56− were seeded at 2500–3000 cells/cm^2^ in proliferation medium. This medium contains 10% FBS (Gibco), DMEM (Gibco), M-199 (Lonza), insulin (Sigma-Aldrich), l-Glutamin (Gibco), Penicillin/streptomycin (Gibco) and growth factors (Peprotech). To obtain myotubes, in CD56+ cell cultures, the medium was replaced by one containing 2% FBS and without growth factors. Myotube cultures were maintained for 10 days, with changes of medium every two days.

### Skin fibroblast isolation and culture

On collection, skin samples were immersed in RPMI 1640 medium and 2% penicillin (1000 U/mL)/streptomycin (10,000 µg/µL) (Gibco, Thermo Fisher Scientific). Then, skin fragments were placed on a moistened surface with Modified Eagle Medium (MEM) (Gibco, Thermo Fisher Scientific), 13% Newborn Calf Serum (Gibco, Thermo Fisher Scientific), 0.4% penicillin/streptomycin and 2 mM l-Glutamine (Sigma-Aldrich, San Luis, MO, USA) and incubated at 37 °C, with 5% CO_2_. Subsequently, fibroblasts (2500–3000 cells/cm^2^) were cultivated in DMEM, 10% FBS and 2% penicillin/streptomycin and once they reached confluence, the cells were recovered by scraping, and the total RNA was purified following the TRIZOL standard protocol.

### Administration of LiCl

LiCl was purchased from Sigma-Aldrich (L7026) and was administered to myoblasts and myotubes at differentiation day 8 at a dose of 10 mM. After 48 h of treatment, RNA and proteins were extracted and mitochondrial and glycolytic function assays were conducted.

### RNA extraction from myoblasts/myotubes, CD56− cells, skin fibroblasts and blood

RNA extraction from these cell types was performed with a RNeasy Mini Kit (QIAGEN). These samples were stored at -80 °C until use.

### Quantitative real-time PCR

The isolated RNA was reverse-transcribed to first-strand complementary DNA (cDNA) using a High-Capacity cDNA Reverse Transcription Kit (Applied Biosystems), according to the manufacturer’s instructions. A total of 25 ng of cDNA was added to SYBR Green Master Mix (Sigma-Aldrich) or TaqMan Gene Expression Master Mix (Thermo Fisher) at a final volume of 10 μL. To investigate the levels of expression of the differentially expressed genes, SYBR Green and primers for *RORA* (NM_002943), *SLC16A1* (NM_001166496), *TFRC* (NM_001128148) and *GAPDH* (NM_002046) were used (Sigma-Aldrich). For the study of myogenesis and CD56− cell expression, TaqMan quantitative RT–PCR assays were performed and *CD9* (Hs00233521_m1), *FRZB* (Hs00173503_m1), *FN1* (Hs00365052_m1), *ITBB1BP2* (Hs00183746_m1), *MYH3* (Hs01074230_m1), *MYOM3* (Hs00537054_m1), *MYOD1* (Hs00159528_m1), *MYOG* (Hs01072232_m1) and *GAPDH* (Hs99999905_m1) probes were used (Thermo Fisher Scientific). These studies were carried out using ‘CFX384 Real-Time PCR Detection System’ (Bio-Rad).

TaqMan quantitative RT–PCR assays were performed using the 7900 HT Fast Real-Time PCR System (Applied Biosystems). A total of 150 ng of cDNA was added to TaqMan® Gene Expression Master Mix 1X (Life Technologies) at a final volume of 100 μl. Statistical analysis was performed using GraphPad Prism v8.01 (GraphPad Software, www.graphpad.com). *p*-values lower than *p* < 0.05 were considered statistically significant for all analysis. Custom-designed TaqMan Low-Density Arrays (TLDAs) (Applied Biosystems) were used to test a series of 63 genes, with selected candidate genes a priori on the basis of an earlier expression profiling study performed using microarrays. This selection was made based on the validation of some of the 74 genes that were found to be deregulated in the muscle of LGMDR1 patients [41]. This selection included genes that showed deregulation in various different biological processes. Genes coding for collagens and fibronectin (which interacts with integrins) were analyzed because they are markers of fibrosis. The TLDAs were used following the protocol recommended by the manufacturer, and the expression of all transcripts was determined relative to the internal housekeeping gene in the TLDAs, GAPDH, for which no alterations in expression were detected. The data analysis of the qRT-PCR results was performed using *SDS2.2.2* program and *RQ manager* v1.1 (*Life Technologies*) to analyze triplicates. Subsequently, calculation of fold-change and identification of significant differences by applying the false discovery rate (FDR) of Benjamini–Hochberg was performed using *RealTime StatMiner* (*Intergenomics*).

### Muscle tissue and cell preparation for western blot analysis

Protein extraction from muscle tissues and cell cultures, as well as the procedure for Western Blot analysis was performed as previously described elsewhere [42]. The used antibodies are detailed in Additional file 5: Table S2. Immunoreactive bands were detected using the SignalFire Plus ECL Reagent (Cell Signaling Technology) and analyzed using the iBright (Invitrogen) image system. Values are given as mean ± Standard Error of the Mean (SEM). Analysis of statistical significance of differences in measurements between patients and controls was performed by parametric Student’s t-test (GraphPad Prism v8.01). Statistical significance was considered when *p* < 0.05 (*p < 0.05, **p < 0.005).

### Immunofluorescence of CD56− cells

CD56− cells grown on coverslips were fixed with 4% paraformaldehyde (Electron Microscope Sciences) for 10 min. Then, they were washed in PBS and permeabilized by addition of 0.2% of Triton-X (Sigma-Aldrich) in PBS with 1% BSA (Biowest) for 10 min and then blocked in a solution containing 1% BSA. For the immunostaining, fixed cells were incubated with the primary antibody overnight at 4ºC. After several washes with PBS, they were incubated with the corresponding secondary antibody for 1 h at room temperature. The solutions for primary and secondary antibodies contained 1% BSA in PBS. Cells were further washed with PBS and coverslips mounted on glass slides in a drop of ProLong mounting medium with DAPI (Life Technologies). The primary antibody used was (monoclonal mouse) anti-TE-7 at a dilution of 1:100 (CBL271), and the secondary antibodies used were goat antimouse conjugated to Alexa-Fluor 488 at a dilution of 1:500 (Life Technologies). For myonuclei, the same myotubes were double stained with DAPI to visualize the nuclei.

### Study of the metabolic function

The metabolic study was performed using a Seahorse XF96 Analyzer (Agilent). Cells were plated in a XF96-well cell culture microplate (Agilent) at a density of 1.5 × 10^4^ cells per well for myoblasts and 4 × 10^4^ cells per well for myotubes. Differentiation was induced by same procedure as described above, and assays were performed at days 10–11 of differentiation, depending on the treatment. Seahorse plates were pre-coated with collagen (Corning) at 50 µg/mL concentration. Data analyses were performed using six technical replicates for each sample.

#### Mitochondrial function

For the study of mitochondrial function, oxygen consumption rates (OCRs) in myoblasts and myotubes were analyzed. One hour before the assay, myoblasts and myotubes were incubated at 37 °C without CO_2_ in unbuffered Basal Assay Medium (Agilent) supplemented with 10 mM glucose, 2 mM l-glutamine and 1 mM sodium pyruvate, pH 7,4. OCR was monitored over time, before and after injections of mitochondrial inhibitors oligomycin (1 μM), proton ionophore fluorocarbonyl cyanide phenylhydrazone (FCCP, 1 μM) and Rotenone/Antimycin (0.5 μM); inhibitor of ATP synthase, mitochondrial oxidative phosphorylation uncoupler and inhibitors of mitochondrial complex I and complex III, respectively. Crystal violet was used for data normalization, reading the optical density at 595 nm. Data collection and analyses were performed using Wave software (Version 2.6, Agilent). Bioenergetic and mitochondrial function parameters such as basal respiration, maximal respiration, ATP production, spare respiratory capacity (*Maximal Respiration*/*Basal Respiration* × 100) and coupling efficiency (*ATP Production Rate*/*Basal Respiration Rate* × 100) were analyzed.

#### Glycolytic function

For the study of glycolytic function, extracellular acidification rates (ECARs) in myoblasts and myotubes were analyzed. One hour before the assay, both cellular models were incubated at 37 °C without CO_2_ in unbuffered Basal Assay Medium (Agilent) supplemented with 2 mM l-glutamine (pH 7,4). ECAR was measured over time following injections of glucose (10 mM) to activate glycolysis, oligomycin (1 μM) to inhibit ATP synthase and 2-desoxi-D-glucose (2-DG at 50 mM) to inhibit glycolysis. Crystal violet was used for data normalization, reading the optical density at 595 nm. The data collection and analyses were also performed using the Wave software (Version 2.6, Agilent). Glycolytic function was analyzed based on parameters such as glycolysis, glycolytic capacity and glycolytic reserve.

#### Culture media analysis

One mL of culture media obtained from myoblast and myotube cultures (at day 11 of differentiation) of two controls and two patients were analyzed. Lactate, pH, Ca^+2^ and glucose levels were determined using GEM Premier 4000 analyzer. Ammonium, lactate dehydrogenase and calcium levels were determined using Cobas 8000–702 Roche.

### Supplementary Information

**Additional file 1: Fig. S1.** PGC1α protein expression analysis in muscle samples. Western blot and densitometry analysis. Error bars represent standard error of the mean (SEM).

**Additional file 2: Fig. S2.** Mitochondrial function in myoblasts and in myotubes at day 10 of differentiation. Mitochondrial function **A** in myoblasts and **B** in myotubes at day 10 of differentiation. Basal respiration, maximal respiration, ATP Production and coupling efficiency **C** in myoblasts and **D** myotubes.

**Additional file 3: Fig. S3.** Glycolytic function in myoblasts and in myotubes at day 10 of differentiation. Glycolytic function analyzed **A** in myoblasts and **B** in myotubes at day 10 of differentiation. Glycolysis, Glycolytic capacity and Glycolytic reserve **C** in myoblasts and **D** myotubes.

**Additional file 4: Table S1.** Fold-change values of the gene expression analysis in tissue and cells in culture. Shaded in green downregulated genes and shaded in red upregulated genes. *Results previously published by our group [42]. N.d.: Not detected. X: Not analysed.

**Additional file 5: Table S2.** Used antibodies.

## Data Availability

All data that support the findings of this study are included within this paper and its Supplementary Information files.
