# Supplementary figures and images for: Altered expression of proteins involved in metabolism in LGMDR1 muscle is lost in cell culture conditions
Source: Orphanet J Rare Dis. 2023 Oct 10;18:315. doi: 10.1186/s13023-023-02873-5 (PMC10565977; doi:10.1186/s13023-023-02873-5)

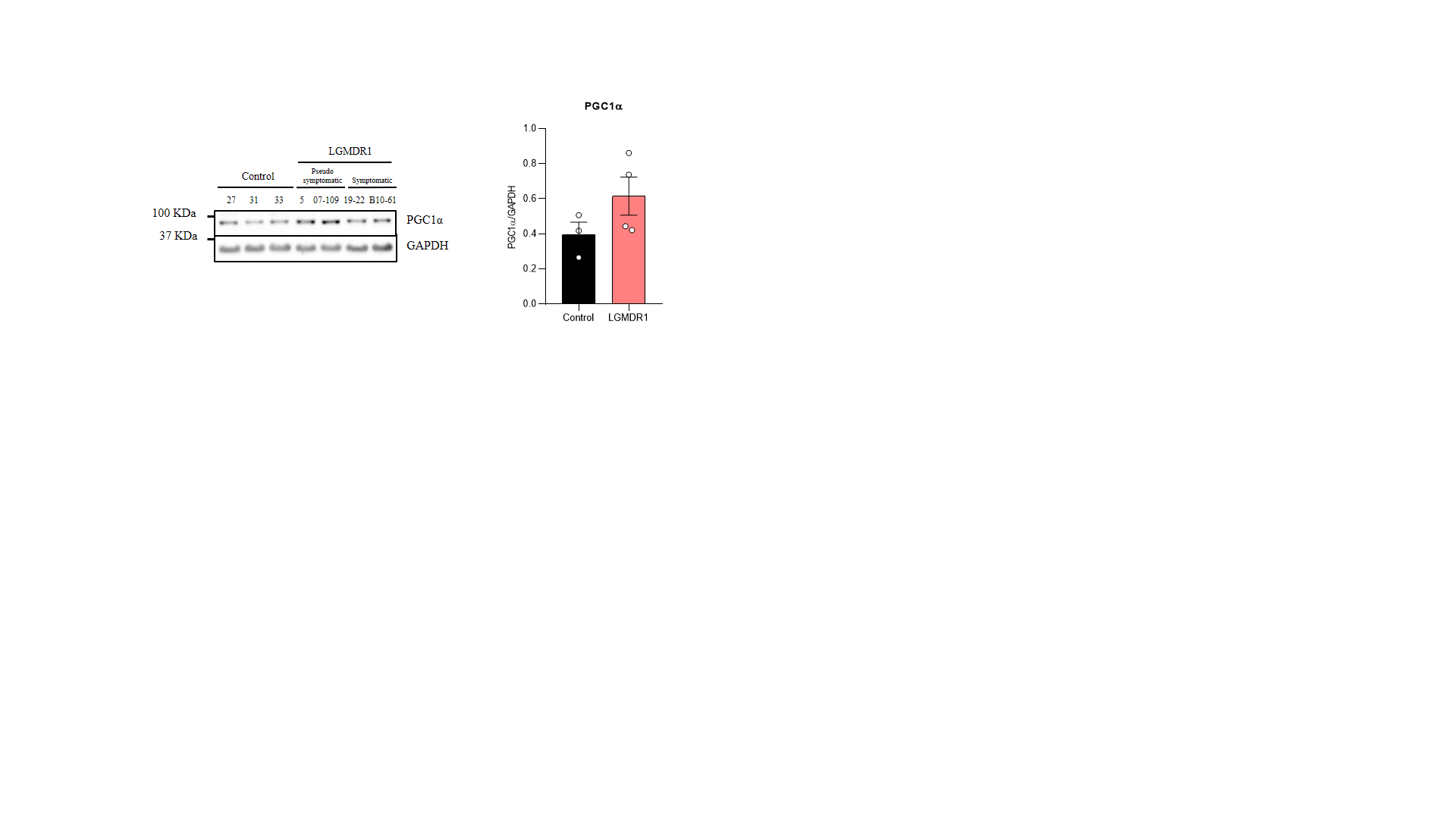

Supplement: Supplementary file 1 — Additional file 1: Fig. S1. PGC1α protein expression analysis in muscle samples. Western blot and densitometry analysis. Error bars represent standard error of the mean (SEM). [file 13023_2023_2873_MOESM1_ESM.tif]

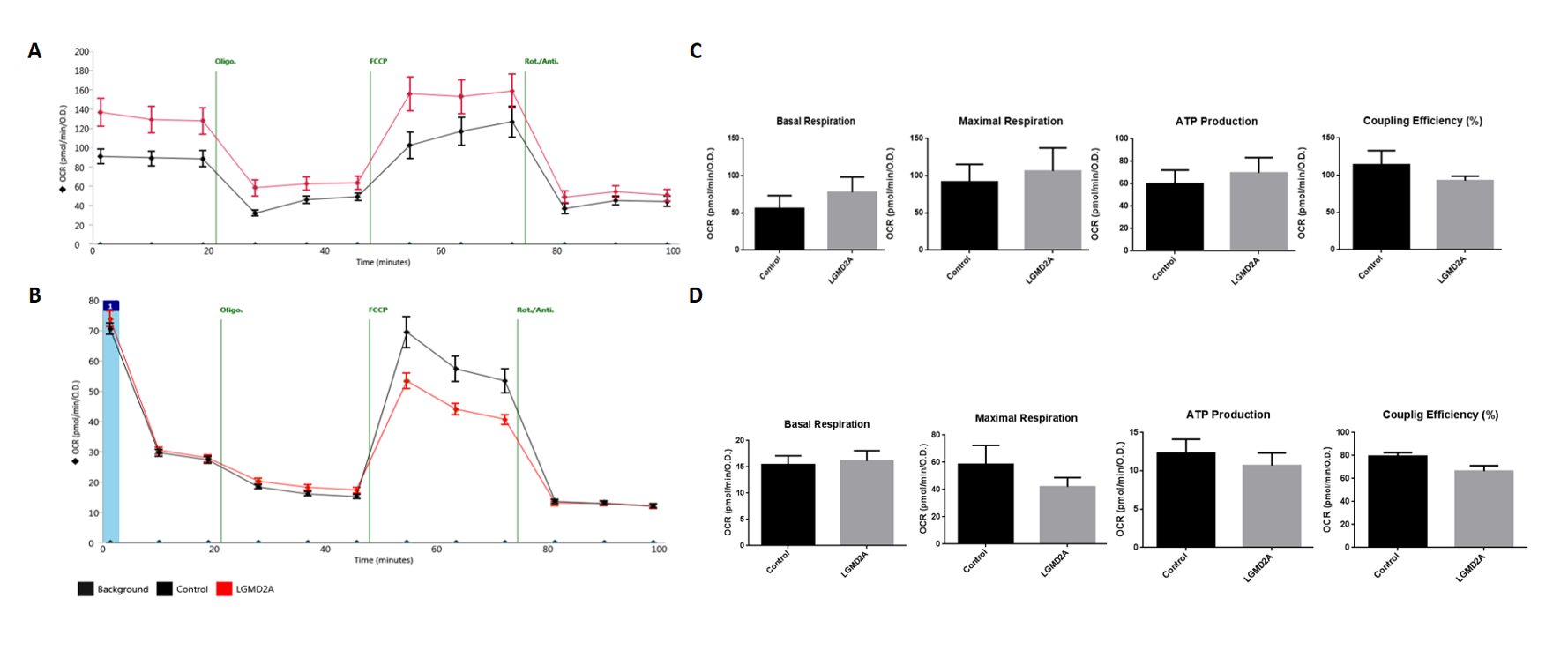

Supplement: Supplementary file 2 — Additional file 2: Fig. S2. Mitochondrial function in myoblasts and in myotubes at day 10 of differentiation. Mitochondrial function A in myoblasts and B in myotubes at day 10 of differentiation. Basal respiration, maximal respiration, ATP Production and coupling efficiency C in myoblasts and D myotubes. [file 13023_2023_2873_MOESM2_ESM.tif]

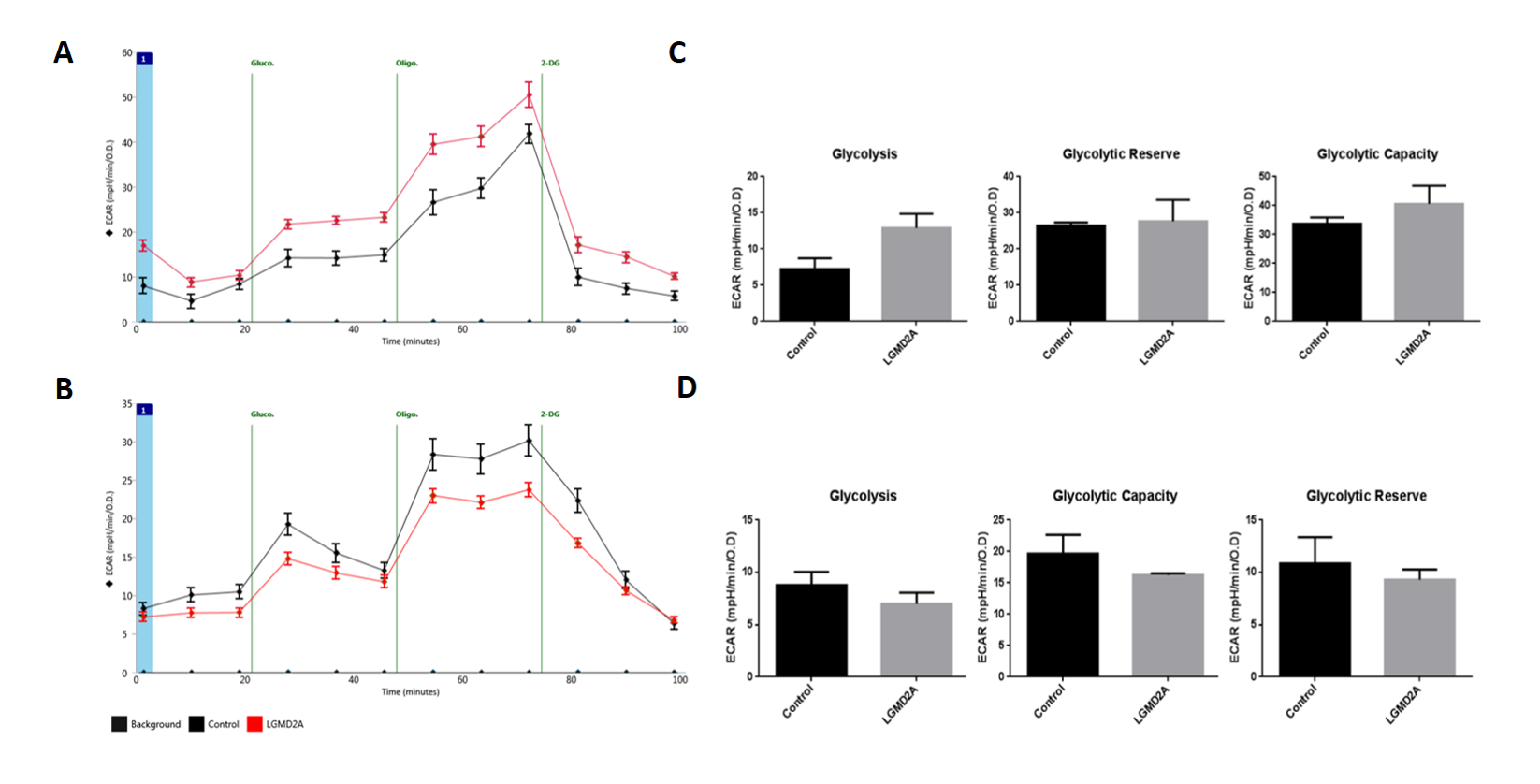

Supplement: Supplementary file 3 — Additional file 3: Fig. S3. Glycolytic function in myoblasts and in myotubes at day 10 of differentiation. Glycolytic function analyzed A in myoblasts and B in myotubes at day 10 of differentiation. Glycolysis, Glycolytic capacity and Glycolytic reserve C in myoblasts and D myotubes. [file 13023_2023_2873_MOESM3_ESM.tif]
