# Supplementary material for: Altered expression of proteins involved in metabolism in LGMDR1 muscle is lost in cell culture conditions
Source: Orphanet J Rare Dis. 2023 Oct 10;18:315. doi: 10.1186/s13023-023-02873-5 (PMC10565977; doi:10.1186/s13023-023-02873-5)
Supplement: Supplementary file 5 — Additional file 5: Table S2. Used antibodies. [file 13023_2023_2873_MOESM5_ESM.docx]

**Additional table 2**. Used antibodies.

| **Primary Antibody** | **Manufacturer** | **Reference** |
| --- | --- | --- |
| GAPDH | Cell Signaling Technology | #2118 |
| PGC1α | NovusBio | NBP1-04676 |
| MCT1 | Sigma-Aldrich | HPA071055 |
| Hexokinase II | Santa Cruz Biotechnology | sc-374091 |
| SFRP4 | Invitrogen | PA5-52679 |
| GLUT5 | Invitrogen | MA1-036 |
| Akt | Cell Signaling Technology | #9272 |
| P-Akt | Cell Signaling Technology | #4060 |
| **Secondary Antibody** | **Manufacturer** | **Reference** |
| Anti-rabbit IgG, HRP-linked  Anti-mouse IgG, HRP-linked | Cell Signaling Technology  DAKO | #7074  P0260 |
